# CAR-T therapy-based innovations in the enhancement of contemporary anti-tumor therapies

**DOI:** 10.3389/fimmu.2025.1622433

**Published:** 2025-07-02

**Authors:** Wan-Ying Zhang, Lang-Yu Yang, Xing-Xing Fan

**Affiliations:** Dr. Neher’s Biophysics Laboratory for Innovative Drug Discovery, State Key Laboratory of Quality Research in Chinese Medicine, School of Chinese Medicine, Macau University of Science and Technology, Macau, Macau SAR, China

**Keywords:** car-t, tumor microenvironment, artificial intelligence, tumor, immunity

## Abstract

Chimeric antigen receptor T (CAR-T) cell therapy has revolutionized the treatment landscape for hematologic malignancies; however, its efficacy in solid tumors remains limited due to antigen heterogeneity, a suppressive tumor microenvironment, and tumor-intrinsic resistance mechanisms. In parallel, immune checkpoint blockade (ICB) therapies have achieved clinical milestones but often fail due to impaired antigen presentation, interferon signaling dysregulation, and immune exclusion. Recent advances in CAR-T therapy-based technologies including multi-specific and armored CAR constructs, gene-editing strategies, and synthetic circuits offer new opportunities to overcome these barriers and expand therapeutic efficacy. Artificial intelligence (AI) has further accelerated the discovery of novel tumor antigens, optimized CAR design, and enabled real-time modeling of treatment responses. Integration of CAR-T therapy with AI-driven platforms, metabolic reprogramming, bispecific antibodies, and advanced single-cell analytics represents a powerful strategy to enhance tumor targeting and durability of response. This review summarizes emerging CAR-T therapy-based innovations, explores their synergistic applications with immunotherapies, and discusses current challenges related to safety, manufacturing, cost, and biomarker validation. These multidisciplinary efforts collectively pave the way toward more effective and personalized cancer treatment.

## 1 Introduction

In recent years, immunotherapy has emerged as a recognized treatment modality following chemotherapy and targeted therapy (1). The development of novel immune modulators, including immune checkpoint blockade (ICB), adoptive cell therapies, and bispecific antibodies, has significantly enhanced the precision and diversity of anti-tumor strategies by activating or reprogramming the host immune response (2–4). Compared to traditional approaches such as chemotherapy, radiotherapy, and targeted therapy, immunotherapy offers advantages not only in its high specificity for tumor-associated antigens but also in inducing durable responses through immunological memory and demonstrating adaptive regulation potential for metastatic and drug-resistant tumors (5). Recent data indicated that globally approved immunotherapeutic agents now span multiple categories, including checkpoint inhibitors, cell-based therapies, and other innovative immunomodulators with indications across a range of solid and hematologic malignancies (6–9).

Despite these advances, the efficacy of immunotherapy in solid tumors remains limited by several biological and immunological barriers. Key challenges include antigen heterogeneity, stromal exclusion, T cell exhaustion, and suppressive metabolic conditions that collectively impair immune cell infiltration and cytotoxic function (10). These barriers set the stage for therapy resistance and highlight the complexity of designing effective immunotherapeutic strategies in solid tumors.

However, in clinical practice, low response rates and high relapse rates remain significant obstacles, making therapeutic resistance a persistent challenge. As highlighted by Chen et al., the limited success of Chimeric antigen receptor T (CAR-T) therapy in solid tumors stems from a combination of inherent challenges, including diverse antigen expression, the presence of a profoundly immunosuppressive tumor microenvironment, and toxicity-related limitations. Together, these barriers complicate efforts to attain long-lasting clinical benefit (11). Notably, despite the widespread clinical use of anti-programmed death protein 1 (αPD-1) inhibitors in combination with conventional chemotherapy, five-year survival rates often remain below 10% (12). In most solid tumors, such as pancreatic cancer and glioblastoma, the response rate to ICB monotherapy is less than 20%, primarily due to antigen presentation defects and insufficient immunogenicity caused by tumor heterogeneity (13–15). Even among initial responders, more than 65% relapse within two years due to acquired resistance. Resistance mechanisms involve a dynamic interplay between tumor-intrinsic adaptations and microenvironmental remodeling (16). Moreover, tumor heterogeneity and immunosuppressive microenvironments continue to impede technological breakthroughs (17, 18).

These widely recognized limitations in solid tumors including antigenic heterogeneity, immune exclusion by dense stroma, and sustained T cell dysfunction form the conceptual basis for improving CAR-T cell strategies beyond hematologic cancers (19). Rather than relying on conventional CAR constructs alone. Recent studies have explored modifications such as dual antigen targeting, co-stimulatory enhancements, and engineering of resistance to immunosuppressive signals (20). For example, armored CAR-T cells designed to secrete pro-inflammatory cytokines or resist exhaustion-related pathways are being evaluated as means to improve infiltration and persistence within the tumor microenvironment (21). Localized delivery of CAR-T cells and combination therapies involving checkpoint blockade are also under investigation as routes to overcome poor immune accessibility and functional suppression (22). These approaches not only aim to address the low response and high relapse rates documented in clinical trials but also represent a shift toward personalized, mechanism-guided CAR-T design.

In this review, we systematically analyzed the mechanisms underlying resistance to immunotherapy and highlighted sophisticated CAR-T therapy-based strategies, including multidimensional combinatorial approaches designed to overcome these barriers, offering new insights to accelerate clinical translation and advance personalized cancer treatment.

## 2 Mechanisms of resistance to immunotherapy and associated challenges

### 2.1 Tumor-intrinsic mechanisms

#### 2.1.1 Insufficient tumor mutational burden

Analyzing and addressing tumor-intrinsic mechanisms is essential for understanding resistance to immunotherapy. One of the primary challenges is insufficient tumor mutational burden, which has gained significant attention as a predictive biomarker in recent years (23). TMB refers to the total number of somatic mutations accumulated in the tumor genome (24). A high TMB can lead to the generation of more neoantigens, which facilitates T cell recognition and tumor cell elimination (25). However, in many tumors, few neoantigens are expressed, and tumor cells often resemble normal tissue too closely for T cells to effectively distinguish them. As a result, immune checkpoint inhibitors exhibit limited efficacy, contributing to immune resistance. To address this issue, recent studies have proposed using Deoxyribonucleic Acid (DNA)-damaging agents to increase tumor mutational rates, thereby enhancing sensitivity to immunotherapy (26, 27). Although these approaches are still in early stages, they present novel avenues for therapeutic development. A clinical study published in *The Lancet* demonstrated the potential of mRNA-4157 (V940), a personalized neoantigen peptide vaccine designed via individualized sequencing, in combination with Keytruda for melanoma. By targeting a small number of existing mutations, this vaccine enhanced immune responses and reduced recurrence risk and mortality. The study is currently in phase II clinical trials NCT03897881 (28).

In addition to enhancing neoantigen load, epigenetic modulation has emerged as another approach to address low TMB. DNA methylation inhibitors, such as decitabine, can activate endogenous retroviral elements, thereby mimicking neoantigen effects. In ovarian cancer models, decitabine combined with immune checkpoint blockade has been shown to increase T cell infiltration by threefold. Orsulic et al. demonstrated in a mouse model that the combination of decitabine and Cytotoxic T-Lymphocyte–Associated Protein 4 (CTLA-4) blockade significantly enhanced lymphocyte migration and function. In contrast, Keathley et al. investigated this approach in patients and observed epigenetic remodeling of immune pathways following decitabine treatment, supporting its clinical relevance (29, 30). With the advancement of big data models, TMB prediction algorithms have also contributed to solving this issue. AI-assisted models can identify patients with “hidden high TMB” even if the overall TMB is low, thereby uncovering additional beneficiaries. For example, Wang et al. proposed a decision-classification model called TMBserval, which enables fine-grained patient stratification with high operability and clinical applicability (31). Similarly, a radiomics-based machine learning model using features extracted from enhanced abdominal CT images has been developed to predict TMB status in gastric cancer (32).

#### 2.1.2 Defects in antigen presentation

Effective T cell recognition of tumor cells typically requires two essential components: the processing of tumor antigens and their presentation on the cell surface via major histocompatibility complex class I (MHC-I) molecules (33). Defects in antigen presentation may arise from the downregulation or loss of MHC-I expression or from mutations or functional inactivation of key components in the antigen processing and loading machinery, such as Transporter Associated with Antigen Processing (TAP) or β2-microglobulin (B2M) (34). In such scenarios, tumor cells become “invisible” to T cells, thereby rendering immunotherapies ineffective. To restore MHC-I expression, small-molecule agents such as histone deacetylase (HDAC) inhibitors have been investigated (35). Recent studies have demonstrated that the HDAC inhibitor OBP-801 can upregulate the expression of the immunoproteasome subunit (Low Molecular Weight Protein 2) LMP2, thereby enhancing MHC-I-mediated antigen presentation. Notably, Narukawa et al. showed that OBP-801, when used in combination with anti-PD-1 antibody, significantly improved the anti-tumor effect in clear cell renal cell carcinoma models, supporting its role in enhancing immune checkpoint blockade therapy (36). In addition to pharmacological modulation, alternative cytotoxic mechanisms can also be leveraged to bypass defective antigen presentation. Natural killer (NK) cells, which can target tumor cells lacking MHC-I, have shown promise in this regard. CAR-NK therapies have achieved notable progress in both preclinical and clinical settings (37). For instance, in a phase I/IIa clinical trial, umbilical cord blood-derived CD19-targeting CAR-NK cells were used to treat patients with relapsed or refractory non-Hodgkin lymphoma (NHL) and chronic lymphocytic leukemia (CLL). Among the 11 patients enrolled, 8 (73%) responded to the therapy, with 7 achieving complete remission. Importantly, no major toxicities were observed, and CAR-NK cells persisted *in vivo* for up to one year (38) NCT03056339. Beyond hematologic malignancies, CAR-NK cells have also demonstrated therapeutic potential in solid tumors. For example, Glypican-3 (GPC3)-targeted CAR-NK cells exhibited specific cytotoxicity against GPC3-expressing hepatocellular carcinoma cells in both *in vitro* and *in vivo* models, showing resistance to immunosuppressive factors in the tumor microenvironment (39). Furthermore, preclinical studies have shown promising results for CAR-NK therapies in ovarian cancer and pancreatic cancer (40, 41).

#### 2.1.3 Defects in interferon signaling pathways

Interferons, particularly interferon-gamma (IFN-γ), are critical cytokines secreted by activated T cells. They play essential roles in upregulating MHC-I expression, recruiting immune cells, and directly inhibiting tumor proliferation (42). Defects in the interferon signaling pathway are typically caused by mutations in the IFN-γ receptor, deletions or dysfunction of Janus Kinase (JAK)1/2, or abnormalities in downstream effectors such as Signal Transducer and Activator of Transcription 1 (STAT1) and Interferon Regulatory Factor 1 (IRF1) (43, 44). As a result, tumor cells become unresponsive to immune attacks, ultimately leading to resistance against immunotherapy (45). To overcome these defects, researchers have explored the activation of innate immune pathways to partially compensate for impaired interferon responses. The stimulator of interferon genes (STING) pathway has emerged as a critical regulator of innate immunity. Its activation induces IFN production, thereby enhancing antitumor immune responses (46). STING agonists have been tested in combination with PD-1 blockade in clinical trials for resistant tumors. MK-1454, an intratumorally administered small-molecule STING agonist, activates the STING–TBK1–IRF3 axis to promote Interferon-beta (IFN-β) secretion and augment antitumor immunity (47). Clinical trials targeting lymphoma (NCT03010176) and head and neck squamous cell carcinoma (NCT04220866) have been completed. GSK3745417, a non-cyclic dinucleotide (non-CDN) STING agonist, was developed to overcome the limitations of intratumoral delivery through intravenous administration (48). However, results from a phase I trial showed limited efficacy in treating relapsed or refractory myeloid malignancies and raised significant safety concerns, including systemic inflammatory responses. GSK announced the termination of this program in 2024, citing an unfavorable risk benefit profile (NCT05424380). Preliminary studies have also suggested that small-molecule agents may indirectly restore interferon signaling by modulating JAK–STAT activity. Researchers at Harvard Medical School demonstrated that the Phosphoinositide 3-kinase gamma (PI3Kγ) inhibitor eganelisib suppresses phosphorylation of p21-activated kinase 1 (PAK1), thereby disrupting its interaction with Ras-related C3 botulinum toxin substrate 1(RAC1) and indirectly inhibiting STAT3 activation. This mechanism was particularly effective in acute leukemia with high expression of PIK3R5 (a regulatory subunit of PI3Kγ), restoring T cell surveillance (49). More recently, interferon-deficient tumor cells have been recognized as sources of novel neoantigens, potentially targetable by personalized T Cell Receptor-Engineered T cells (TCR-T) cell therapy. In a phase I clinical trial, Borgers et al. demonstrated that a novel TCR-T therapy, BNT221, led to a 20% reduction in metastatic melanoma burden within six weeks (NCT04625205) (50). Future development in this field is expected to focus on predicting T cell recognition efficiency against IFN-deficient tumor neoantigens and building cost-effective TCR-T platforms (51, 52).

### 2.2 Immunosuppressive tumor microenvironment

#### 2.2.1 T cell exhaustion

As one of the most difficult barriers to overcome in immunotherapy resistance, T cell exhaustion within the immunosuppressive tumor microenvironment (TME) plays a pivotal role. This dysfunctional state arises from chronic antigen stimulation especially in the context of tumors and is characterized by progressive loss of effector function, sustained expression of inhibitory receptors, and metabolic dysregulation (53). It is characterized by reduced cytotoxic activity, sustained expression of inhibitory receptors, and decreased secretion of effector cytokines such as IFN-γ and Tumor Necrosis Factor-alpha (TNF-α). Current evidence suggests that exhausted T cells are often refractory to conventional ICB, limiting therapeutic efficacy.

To address this challenge, considerable efforts have been made to develop next-generation checkpoint inhibitors targeting molecules beyond PD-1, such as T-cell immunoglobulin and mucin-domain containing-3 (TIM-3), Lymphocyte Activation Gene-3 (LAG-3), and T cell immunoreceptor with Ig and ITIM domains (TIGIT) (54). TIM-3 is highly expressed on exhausted T cells and acts synergistically with PD-1 to suppress T cell function (55). It also inhibits IFN-γ signaling and promotes regulatory T cell (Treg) activity, thereby exacerbating the immunosuppressive TME (56). Clinical trials investigating the combination of anti-TIM-3 antibodies with anti-PD-1 agents have demonstrated antitumor activity in patients with advanced or metastatic recurrent biliary tract cancer (57, 58). LAG-3 inhibits T cell activation by binding to MHC II molecules (59). Relatlimab, the first anti-LAG-3 antibody, in combination with nivolumab (anti-PD-1), has received FDA approval for metastatic melanoma and significantly prolongs progression-free survival (PFS) (60). In murine models, LAG-3 blockades have been shown to restore CD8^+^ T cell metabolic adaptability and reduce the expression of exhaustion markers such as PD-1 and TIM-3 (61). TIGIT suppresses T cell and NK cell activity through competitive binding with CD155 (PVR) and facilitates Treg-mediated immunosuppression (62). Preclinical studies have demonstrated that TIGIT antibodies (e.g., tiragolumab) combined with PD-L1 blockade can significantly enhance antitumor immune responses (63). Nevertheless, emerging checkpoint inhibitors may induce systemic inflammatory responses, such as cytokine release syndrome, highlighting the need for dynamic monitoring systems to assess toxicity in real time (64). T cell reprogramming is another key strategy, which aims to partially reverse exhaustion through metabolic activation (e.g., mTOR modulators) (65, 66) or epigenetic intervention (e.g., HDAC inhibitors) (67). However, clinical translation remains challenging. Some metabolic regulators exhibit systemic toxicity, necessitating optimized dosing strategies or the development of tissue-specific delivery vehicles. Off-target effects of epigenetic drugs also require precision regulation through gene-editing technologies (68).

Increasing the proportion of memory T cells is another promising approach, which typically involves enhancing the initial activation quality of T cells to promote the formation of long-lived memory populations and sustain immune surveillance. Techniques such as metabolic reprogramming (e.g., glycogen and ketone metabolism) (69), epigenetic interventions (e.g., β-hydroxybutyrylation and ACLY inhibition) (69), CD4^+^ T cell help, and optimized antigen stimulation have been shown to boost memory T cell populations and mitigate exhaustion (70, 71).

In addition to inhibitory receptor upregulation, the downregulation of costimulatory molecules such as the tumour necrosis factor receptor OX40 (CD134) is a critical feature of T cell dysfunction within the TME. OX40, a member of the tumor necrosis factor receptor superfamily, provides essential costimulatory signals that enhance T cell proliferation, survival, and cytotoxic function (72, 73). Chronic antigen exposure in the TME leads to decreased OX40 expression on exhausted T cells, contributing to impaired effector responses and sustained exhaustion (74).

Therapeutic interventions aimed at restoring OX40 signaling have demonstrated promising antitumor effects. Agonistic antibodies targeting OX40 can upregulate its signaling pathway, resulting in enhanced T cell cytotoxicity, reversal of exhaustion phenotypes, and promotion of memory T cell formation (72, 73). Preclinical models showed that OX40 stimulation not only reinvigorates CD8^+^ T cells but also reduces regulatory T cell-mediated suppression, thereby remodeling the TME toward a more immunogenic state (74). Clinical trials investigating OX40 agonists, alone or in combination with checkpoint inhibitors such as anti-PD-1, have reported improved tumor regression and durable immune responses (73). These findings highlight the therapeutic potential of targeting costimulatory pathways like OX40 to overcome T cell exhaustion and enhance cancer immunotherapy efficacy.

#### 2.2.2 Accumulation of immunosuppressive cells

Tumors are known to recruit and activate three major types of immunosuppressive cells to evade immune surveillance: Tregs (75), myeloid-derived suppressor cells (MDSCs) (76), and tumor-associated macrophages (TAMs) (77). These cells often accumulate in the TME, forming a formidable immune-exclusion barrier.

To overcome the immunosuppressive effects of TAMs, recent studies have explored the use of engineered macrophages, known as CAR-M (chimeric antigen receptor macrophages). Unlike conventional TAMs that often promote tumor progression, CAR-M can be reprogrammed to phagocytose tumor cells and reshape the tumor microenvironment toward a pro-inflammatory state. Li et al. provided a comprehensive review of CAR-M strategies in solid tumors, highlighting their ability to enhance antigen presentation and stimulate adaptive immune responses (78).

Selective inhibition of Tregs has emerged as a novel therapeutic approach (79). Kong and colleagues reviewed cytokine-based strategies involving engineered fusion proteins such as ALKS 4230. This agent, composed of IL-2 fused with a CD25 domain, selectively activates intermediate-affinity IL-2 receptors (IL-2Rβγ) while avoiding high-affinity receptors (IL-2Rαβγ), thereby preferentially expanding CD8^+^ T cells and NK cells without promoting Treg proliferation (80). In phase I/II clinical trials for ovarian and head and neck cancers, ALKS 4230 demonstrated good tolerability with no observed Treg expansion. When combined with PD-1 inhibitors, it significantly enhanced CD8^+^ T cell infiltration in tumors (80). Neoleukin-2/15, an IL-2 mimetic reengineered via computational biology, binds only the IL-2Rβγ heterodimer, completely avoiding CD25 (IL-2Rα) interaction. In murine colorectal cancer models, it showed antitumor efficacy comparable to native IL-2 with substantially reduced toxicity (81). Moreover, chemokines such as C-C motif chemokine ligand 22 (CCL22) recruit immunosuppressive Tregs to the tumor site via C-C chemokine receptor type 4 (CCR4). Targeting CCR4 can thus prevent Treg trafficking and reverse immunosuppression (82).

Similarly, CCR8 is selectively expressed on Tregs and has emerged as another promising therapeutic target for Treg depletion. Unlike CCR4, which is also expressed on a subset of peripheral Tregs, CCR8 expression is highly restricted to immunosuppressive Tregs within the tumor microenvironment, making it an attractive candidate for selective targeting (83). Preclinical studies including the development of afucosylated anti−CCR8 antibodies such as RO7502175 and JTX−1811, as well as novel candidates like BAY 3375968 have demonstrated that CCR8-targeted depletion efficiently eradicates intratumoral Tregs while sparing peripheral Tregs (84, 85). This selective depletion enhances CD8^+^ T cell and NK cell infiltration, reverses T cell exhaustion, and promotes tumor regression in multiple murine tumor models (86). Mechanistically, it reduces expression of immunosuppressive checkpoint molecules on Tregs and alleviates immune exclusion, enabling synergistic activity with PD−1 blockade (87). These preclinical findings have led to ongoing Phase I clinical trials evaluating anti−CCR8 strategies in solid tumors, underscoring CCR8’s potential as a next-generation target for modulating the immunosuppressive TME (88).

Strategies targeting MDSCs aim to disrupt their development and recruitment (89). The colony-stimulating factor 1 receptor (CSF-1R) pathway is crucial in MDSC differentiation. Inhibition of CSF-1R can prevent monocyte differentiation into monocytic MDSCs (M-MDSCs) and limit their accumulation in the TME (90). Ruxolitinib, a JAK/STAT pathway inhibitor, has been shown to reduce MDSC generation by blocking CSF-1R downstream signaling. Preclinical studies demonstrated that its combination with Programmed Death-Ligand 1 (PD-L1) blockade significantly reduced MDSC infiltration and enhanced CD8^+^ T cell activity in Hepatocellular Carcinoma (HCC) models (90).

Pexidartinib, a CSF-1R inhibitor approved for tenosynovial giant cell tumor, also improved immunotherapy responses in colorectal cancer models by suppressing MDSC differentiation (91). Beyond CSF-1R, targeting STAT3 and (Nuclear factor kappa B) NF-κB signaling has shown promise. Xu’s team found that Galectin-8 activates the STAT3/NF-κB pathway via interaction with Leukocyte Immunoglobulin-Like Receptor Subfamily B Member 4 (LILRB4), promoting M-MDSC expansion. Dual-blockade antibodies targeting Galectin-8 or LILRB4 inhibited M-MDSC generation and restored T cell function (92). In further studies, Xia’s group identified that hepatocellular carcinoma cells promote polymorphonuclear MDSC (PMN-MDSC) expansion through an ETV5–S100A9 feedback loop. Antibodies targeting S100A9 disrupted this axis, reduced MDSC levels, and enhanced PD-L1 inhibitor efficacy (93).

In addition, small molecules targeting immunosuppressive enzymes such as arginase and iNOS have yielded promising results (91).

MDSCs also suppress T cell function by generating reactive oxygen species (ROS) and reactive nitrogen species (RNS), which interfere with T cell receptor signaling and cytotoxic activity. Targeting these by-products using Cyclooxygenase-2 (COX-2) inhibitors has shown promise in restoring T cell function (94). Furthermore, MDSCs secrete immunosuppressive cytokines such as interleukin-10 (IL-10) and transforming growth factor-beta (TGF-β), both of which contribute to T cell anergy and regulatory T cell induction. Therapeutic strategies aimed at neutralizing IL-10 and TGF-β or blocking their signaling pathways may further enhance antitumor immune responses, particularly in combination with immune checkpoint inhibitors (95, 96).

Metabolic interventions are also being explored. Disruption of metabolic dependencies in MDSCs, e.g., using fatty acid oxidation (FAO) inhibitors or regulating glycolysis can suppress their development (90, 91). Combined checkpoint inhibition in clinical settings has also shown preliminary success (93).

#### 2.2.3 Metabolic dysregulation

Immunosuppressive features of the TME are often accompanied by metabolic abnormalities, including hypoxia and lactate accumulation. Current research focuses on two major directions: overcoming hypoxia-induced suppression and intervening in lactate metabolism (97). Hypoxic conditions activate hypoxia-inducible factor 1-alpha (HIF-1α), which promotes the expansion of immunosuppressive cells such as Tregs and MDSCs, while concurrently impairing the function of effector T cells (98). Inhibiting HIF-1α has demonstrated the potential to suppress MDSC differentiation in preclinical models (99). Another promising strategy involves the development of nanoparticle-based oxygen delivery systems to reverse hypoxia-induced immune suppression and MDSC expansion. For example, He and colleagues designed self-delivering micelles that primarily target the transendothelial migration of G-MDSCs. Although the original intent was not oxygen delivery, design principles such as pH/redox-responsive release could be adapted to engineer localized oxygen carriers using materials like perfluorocarbons for future applications (100).

Interventions targeting lactate metabolism aim to either reduce lactate production or inhibit its export from tumor cells, thereby alleviating the immunosuppressive burden of lactic acidosis (101). For instance, a phenylalanine-based polymer (MRIAN) was shown to modulate glucose metabolism in MDSCs, reduce reactive ROS, and indirectly lower lactate levels, thus enhancing T cell function (102). Another study demonstrated that acetate could enhance MDSC-mediated immunosuppression via the Free Fatty Acid Receptor 2 (FFAR2) signaling axis, increasing expression of Arginase 1 (Arg1) and (inducible Nitric Oxide Synthase) iNOS; however, inhibition of monocarboxylate transporter 4 (MCT4) successfully reduced lactate export and suppressed MDSC activity (103).

## 3 Advanced strategies for overcoming immunotherapy limitations by CAR-T therapy

### 3.1 Nanomimetic CAR-T cells improve solid tumor infiltration

The insufficient tumor infiltration of T cells in solid malignancies, primarily due to stromal physical barriers and immunosuppressive chemokine gradients, remains a major therapeutic hurdle (104). Some researchers have observed that the impaired biocompatibility of polyethylene glycol (PEG) and the accelerated blood clearance (ABC) have led to insufficient targeting of CAR-T. Cell membrane-coating of nanoparticles, taking advantage of the excellent biocompatibility and versatile functionality of cell membranes can significantly promote the stability of nanoparticles in physiological conditions, resulting in less leakage of drugs (105). Bionanotechnology inspired by natural biological systems offers innovative strategies to enhance tumor homing and penetration of T cells. Recent advancement includes the development of T cell membrane-coated nanodevices such as CD19-specific CAR membrane, which demonstrated the much better-targeting effect than the homologous tumors cell membrane camouflaging. Additionally, functionalizing CAR-T cell membranes with tumor-specific antigens through covalent conjugation techniques had been shown to facilitate antigen-dependent clustering at NALM-6 tumor cells, significantly augmenting tumor infiltration and activating the immune response (106). To address HER2^+^ lung cancer, a biomimetic nanoplatform including Cisplatin-loaded poly (lactic-co-glycolic acid) (PLGA) nanoparticles and coating them with CAR-T cell-derived membranes from genetically engineered human Jurkat T cells, exhibiting effective tumor infiltration at the site of HER2^+^ lung cancer (107). GPC3, a 580-AA heparin sulfate proteoglycan, is expressed in 75% of HCC samples, but not in healthy liver or other normal tissues. Based on this, GPC3 targeting CAR-T cells were used to prepare CAR-T membranes (CMs) to better recognize the HCC. Then, CM was camouflaging mesoporous silica nanoparticles (MSN) loaded with near-infrared (NIR) dye IR780 (a biodegradable photothermal and imaging agent) to enhance IR780 photothermal therapy. The results illustrated the enhanced tumor targeting ability and synergistic photothermal immunotherapeutic effects of the novel agent (108). Overall, this biomimetic strategy represents a promising avenue for effectively overcoming stromal exclusion and reshaping the immunosuppressive tumor microenvironmental by leveraging natural trafficking mechanisms, offering a dual strategy to amplify therapeutic efficacy.

### 3.2 Engineered CAR-T improves efficiency and affordability

Some research include that fully *in vivo* CAR-T generation is being actively explored with systemic administration of CAR-encoding nanocarriers and viral constructs. *In vivo* generation of CAR-T cells eliminates extensive *ex vivo* culture and could prevent the terminal differentiation of CAR-T cells due to *ex vivo* procedures altogether. Several challenges remain to this approach, including the short plasma half-life of systemically administered carriers and possible non-specific targeting of carriers to off-target cells (109, 110). To accelerate the production of stabilized CAR-T through biomaterials, one research had developed a multifunctional alginate scaffold (MASTER) for rapid *in vivo* reprogramming of T cells (111). Co-delivery of monocytes and retroviral particles encoding CD19 enabled local release of functional CAR-T cells, exhibiting greater persistence than conventional CAR-T cells (111). Pan and colleagues reported a technique addressed the critical bottleneck of low lentiviral transduction efficiency in CAR-T manufacturing by introducing a glycometabolic bioorthogonal engineering strategy. By labeling T cells with azide-glucose to anchor artificial ligands and coating lentiviruses with dibenzocyclooctyne (DBCO) conjugated polyethylenimine (PEI), the platform enabled targeted “click” binding, boosting transduction efficiency to 80% without compromising cell viability (112). Moffett’s group constructed a platform by delivering CAR genes targeting leukemia antigens directly into the nucleus of circulating T cells via polymer nanoparticles, achieving “instant programming *in vivo*” without *in vitro* manipulation. Subsequently, they demonstrated that this strategy induced long-lasting CAR expression in T cells and achieved long-term leukemia remission in mouse models. The modular design of the nanoparticles was found to be suitable for a variety of tumor targets, providing a more efficient and universal solution for generating anti-tumor immunity “on demand” (109). Lipid nanoparticles (LNPs) have been widely used as transfection carriers (113). Billingsley’s team addressed critical challenges in CAR-T cell manufacturing by introducing LNPs as a non-viral, low-toxicity alternative and electroporation for transient CAR mRNA delivery. By synthesizing and screening a library of 24 ionizable lipids, the optimized C14–4 LNP formulation achieved CAR expression levels in primary human T cells comparable to electroporation but with significantly reduced cytotoxicity. Functionally, the resulting CAR-T cells exhibited potent tumor-killing activity against Nalm-6 leukemia cells, matching the efficacy of electroporation-derived cells. This platform eliminated risks of permanent CAR expression from viral vectors and minimized cell damage from harsh electroporation, offering a scalable, cost-effective strategy for safer and more flexible CAR-T production (114).

### 3.3 Real-time monitoring and adaptive feedback in CAR-T therapy

Traditional detection methods have utilized enzymes, transport proteins or membrane proteins to promote the accumulation of radioactive tracers (such as 9-(4- [18 F] fluoro-3 -(hydroxymethyl) butyl) guanine) in glioblastoma. This requires genetic modification of the therapeutic cells, and genetic modification utilizes the previous viral packaging ability, which may lead to immunogenicity and may interfere with its function (115). To enable real-time monitoring of CAR-T cell activity, precise tracking of *in vivo* distribution, and dynamic functional modulation, researchers have integrated nanotechnology into CAR-T therapy, establishing innovative platforms for precision surveillance and closed-loop feedback control (116). Harmsen and colleagues presented a dual-modal nanoparticle platform inculding positron emission tomography (PET) and near-infrared fluorescence (NIRF) for non-genomic labeling of CAR-T cells, enabling real-time, non-invasive tracking of their biodistribution and persistence without compromising viability or cytotoxic function. In an ovarian peritoneal carcinomatosis model, the technology allowed longitudinal whole-body imaging via PET and NIRF, providing critical insights into CAR-T pharmacokinetics to optimize dosing and mitigate off-target risks (117). Magnetic resonance imaging (MRI), with the advantages of non-ionizing radiation, high resolution and multi-tissue contrast, has a crucial role to perform in both clinical diagnosis and research (118). Another research team developed a ferumoxytol-labeled CAR-T cell platform via mechanoporation. Furthermore, iron oxide nanoparticles enabled the photoacoustic imaging (PAT) and magnetic particle imaging (MPI) to track CAR-T cells tumor homing and off-target migration *in vivo*. In a mouse model of *in situ* tibial osteosarcoma, researchers constructed a retroviral vector for expressing anti-B7-H3-4-1BBζ CAR and inserted the BFP sequence downstream of the CD3ζ domain. MRI showed that anti-B7 homologue 3 (B7-H3) CAR-T cells homed to the osteosarcoma site and were distributed to off-target sites such as the spleen and liver after labeling. PAT and MPI further visualized the dynamics of CAR-T cell infiltration within the tumor (119). Xie’s group utilized glucose-coated ultra-small superparamagnetic iron oxide nanoparticles (USPIOs) to label anti-hEGFRvIII/IL13Rα2 CAR-T cells, enabling non-invasive MRI monitoring of their infiltration and persistence in glioblastoma. After injection of USPIO-labeled CAR-T cells, an increase in low signal was detected in the GBM model by magnetic susceptibility-weighted imaging MRI 3 to 14 days after injection. In addition, the presence of NPs and CAR-T cells was confirmed on serial histologic sections. This multimodal imaging approach correlated CAR-T biodistribution with therapeutic response, offering translational tools to optimize solid tumor immunotherapy (120). Nanotechnology methods utilize non-genomic markers - such as dual-mode PET/NIRF nanoparticles, ferulic xylitol or glucose-coated uspio - for external application through techniques such as mechanical processing, thereby eliminating genetic engineering, maintaining the viability and cytotoxicity of CAR-T cells, and avoiding the risk of immunogenicity (121, 122). Crucially, the nano-platform achieves multimodal, real-time, and longitudinal tracking through complementary imaging techniques (PET, MRI, NIRF, PAT, MPI), providing high-resolution insights into whole-body biodistribution, tumor localization, off-target migration, and ionizing radiation-free persistence (123, 124). This allows for the correlation between CAR-T kinetics and therapeutic response, optimizes drug administration safety, and provides closed-loop feedback for immunotherapy of solid tumors, which cannot be achieved by traditional gene labeling methods (125).

### 3.4 Controlled associated cytokine release syndrome and neurotoxicity

One challenge faced by CAR-T therapy is CRS. Studies show that approximately 60% to 80% of patients receiving CAR-T treatment may develop CRS. In CRS, CAR-T cells are activated after binding to tumor cell antigens, continuously expanding and releasing a large amount of cytokines, thereby causing systemic inflammatory responses and even leading to the death of patients (126). To overcome the side effects of CRS associated with CAR-T therapies, some nanoplatform are introduced to suppress these toxicities through cytokine neutralization, targeted immunosuppression, and real-time biomarker monitoring. A lipid nanoparticle system co-delivered the combination gene of interleukin 6 short hairpin RNA (IL-6 shRNA) and CD19-CAR (CAR19 shIL6) targeting leukemia with high CD19 expression. The results showed that antiCD3-LNP/CAR19^+^ shIL6 nanoparticles could induce the generation of stable IL-6 knockdown CAR-T cells within 90 days, exhibiting powerful antitumor capabilities, effectively eradicating Raji tumors while concurrently mitigating CRS in NSG mice. Notably, this approach successfully reduced the CRS associated with CAR-T cell therapy and further improved the convenience of CAR-T usage (127). In another independent research, Jun Wang et al. utilized mimetic virus fusion NVs (FuNVs) as CAR protein carriers to produce CAR-T cells by fusion with t-cell membranes stably *in vivo*. It was found that anti-CD19 CAR-T cells produced with FuNVCAR were effective in killing CD19-positive B lymphoma cells and avoided inducing CRS (128). Polyethylene glycolization (PEG) of CAR-T cells created a transient aggregation barrier that blocked interactions with tumor cells, monocytes and reduces the severity of cytokine-driven CRS. This reversible modification temporarily inhibited excessive tumor lysis and monocyte activation, thereby reducing the release of toxic cytokines (129). In addition, engineered erythrocyte-derived extracellular vesicles (RBC EVs) immobilized with gp350 by electroporation showed enhanced tumor-specific targeting of CD21 B-cell malignancies while minimizing systemic toxicity. By loading chemotherapeutic agents, the EVs selectively delivered cytotoxic payloads to tumor cells, obtaining effective antitumor efficacy without triggering off-target immune activation or cytokine release. This precision reduced the risk of systemic inflammation and CRS-like complications, providing a safer alternative to traditional CAR-T or broad-spectrum immunotherapy (130).

## 4 Recent advances in combining CAR-T therapy with ICB

Given that ICB can restore T cell activity by alleviating functional inhibition, combining CAR-T therapy with immune checkpoint inhibitors has emerged as a promising strategy to overcome the limitations of CAR-T monotherapy, especially in solid tumors (131). The rationale behind this combination involves two synergistic mechanisms: ICB agents relieve the functional suppression of CAR-T cells, while also remodeling the tumor microenvironment to reduce immunosuppression (131). This dual effect enhances the proliferation and persistence of CAR-T cells and delays or reverses their exhaustion and apoptosis, ultimately improving treatment durability and depth of response.

### 4.1 Clinical applications

Building upon the mechanistic rationale outlined above, the combination of CAR-T therapy with immune checkpoint modulation has now progressed from preclinical promise to clinical investigation (22). Various strategies incorporating PD-1/PD-L1 blockade, CTLA-4 inhibition, or co-stimulatory molecule activation (e.g., OX40) are being evaluated in both hematological and solid malignancies (131, 132). These approaches aim to enhance the clinical efficacy of CAR-T therapy by improving T cell infiltration, persistence, and functional resilience within the immunosuppressive tumor microenvironment (133). The following subsections highlight representative clinical applications, relevant trial outcomes, and mechanistic insights for each type of immune modulator when used in combination with CAR-T therapy.

#### 4.1.1 PD-1 antibody combined with CAR-T

PD-1 antibodies block the PD-1/PD-L1 signaling axis, thus lifting the immunosuppressive constraints on T cells. CAR-T cells, on the other hand, exert cytotoxic effects by targeting tumor-specific antigens (134). When combined, these approaches can synergistically enhance CAR-T cell activity and modulate the immunosuppressive tumor microenvironment (135). Representative clinical trials have evaluated the efficacy of combining anti-PD-1 agents (e.g., nivolumab) with CD19-directed CAR-T therapy in patients with relapsed/refractory B-cell lymphomas. Preliminary data indicate a complete response (CR) rate of 67% in the combination group, significantly higher than the 45% observed in the CAR-T monotherapy group, without increasing the risk of severe cytokine release syndrome (CRS) (NCT03085173). Another clinical trial investigated the combination of B-cell Maturation Antigen (BCMA)-targeted CAR-T cells with pembrolizumab in multiple myeloma. The results showed that the duration of response (DOR) was extended to 18 months in the combination group, compared to 12 months with CAR-T alone, suggesting that PD-1 blockade may delay CAR-T exhaustion (NCT04381741).

Moreover, promising outcomes have been observed in solid tumors. For instance, BZD1901 developed by Shanghai Cell Therapy Group is a mesothelin-targeted CAR-T cell therapy engineered to secrete PD-1 antibodies locally. In patients with advanced ovarian cancer, this approach achieved a median progression-free survival of 5 months and an overall survival of 17 months. The local secretion of PD-1 antibodies reprogrammed the tumor microenvironment, enhanced CD8^+^ T cell activity, and exhibited manageable toxicity, Apatinib is an anti-angiogenic drug which has been reported to promote CD8 T cells infiltration and this study showed potential of triple therapy in refractory epithelial ovarian cancer in patients (135). These findings demonstrate that combining PD-1 antibodies with CAR-T therapy can overcome tumor antigen heterogeneity and immunosuppressive barriers, particularly in solid tumors.

#### 4.1.2 CTLA-4 antibody combined with CAR-T

CTLA-4 is a key inhibitory receptor on T cells. Its blockade enhances CD28-mediated costimulatory signals, thereby promoting CAR-T cell proliferation and persistence (136). A study by Carl June’s group revealed that CTLA-4 knockout in CAR-T cells led to a twofold increase in antitumor activity. In a chronic antigen exposure model, CTLA-4-deficient CAR-T cells maintained higher CAR expression levels. CTLA-4 deletion in CD19-CAR-T cells lifts inhibition of CD28 signaling under stress, enhancing proliferation, CAR expression, and anti-tumor efficacy *in vitro* and *in vivo* (137). In murine leukemia models, these modified CAR-T cells showed enhanced tumor clearance and reduced expression of exhaustion markers such as LAG-3 and TIM-3 (138). These findings suggest that CTLA-4 may exert negative regulation on CAR-T cell persistence and effector function through pathways beyond simple costimulation inhibition. For instance, CTLA-4 knockout may reduce CAR-T cell susceptibility to chronic antigen-induced exhaustion by preserving metabolic fitness and maintaining activation thresholds (139). Moreover, the downregulation of exhaustion markers such as LAG-3 and TIM-3 may reflect an indirect effect through sustained CD28 signaling, which is known to modulate T cell differentiation and survival (140).

Although the combination of Imfinzi (anti PD-L1) and tremelimumab (anti CTLA-4), developed by AstraZeneca, has not yet been directly tested alongside CAR-T cell therapy, its demonstrated overall survival benefit in a phase III trial for hepatocellular carcinoma provides valuable clinical evidence that dual checkpoint blockade can effectively reprogram the immunosuppressive tumor microenvironment. Given that immune exclusion and T cell exhaustion are key barriers to CAR-T efficacy in solid tumors, the mechanistic rationale underlying this combination may be applicable in CAR-T contexts as well. At the 5-year follow-up, serious treatment-related adverse events (TRAEs) in the STRIDE group remained at 17.5%, consistent with the primary analysis, while serious non-treatment-related adverse events (non-TRAEs) slightly increased to 24.8% from 23.0% (57).

#### 4.1.3 OX40 co-stimulation combined with CAR-T

Incorporation of OX40 co-stimulatory signaling into CAR-T cells has emerged as a promising strategy to overcome the major limitations of CAR-T therapy, particularly in solid tumors. The combination of CAR and OX40 signaling enhances T cell proliferation, persistence, and cytotoxicity, as demonstrated *in vitro* and in mouse models of B cell lymphoma (141). Beyond hematologic malignancies, this approach holds strong potential across a broad range of solid tumors where immunosuppressive microenvironments and T cell exhaustion severely compromise CAR-T efficacy. For instance, in non-small cell lung cancer (NSCLC), OX40 signaling has been shown to reinvigorate exhausted T cells and improve their effector function within highly suppressive tumor contexts (142). In breast cancer, where dense stromal architecture and TGF-β-driven immunosuppression limit immune cell infiltration, OX40 activation enhances CAR-T cell metabolic resilience and persistence, improving their capacity to exert cytotoxic effects (143). Similarly, in pancreatic ductal adenocarcinoma (PDAC), a tumor defined by extensive fibrosis and myeloid-derived suppressor cell infiltration, OX40 stimulation promotes T cell infiltration and reshapes the tumor microenvironment in favor of immune activation (144). In glioblastoma, OX40 engagement has been reported to improve T cell survival and function in the central nervous system despite its highly immunosuppressive milieu (145). Moreover, OX40 signaling may synergize with immune checkpoint blockade or other modulatory agents to further amplify antitumor responses (146). Collectively, these findings suggest that integrating OX40 co-stimulation into CAR-T platforms provides a versatile and promising avenue to enhance therapeutic outcomes across diverse tumor types, warranting further exploration in preclinical and clinical settings.

### 4.2 Molecular engineering innovations

To enhance CAR-T cell efficacy within immunosuppressive TME, molecular engineering approaches such as CRISPR/Cas9-mediated gene editing have been employed. One strategy involves knocking out the PD-1 gene in CAR-T cells to eliminate inhibitory PD-1/PD-L1 signaling and improve CAR-T cell persistence and cytotoxicity in hostile immune contexts (147, 148). For example, Bioheng Biotech has developed a proprietary Quikin CAR-T^®^ platform, which uses CRISPR/Cas9 to simultaneously knock out the PD-1 gene and insert the CD19-CAR construct at the PD-1 locus. This one-step integration strategy effectively abrogates PD-1 expression while ensuring stable CAR expression, leading to enhanced B cell non-Hodgkin lymphoma (B-NHL)- killing activity (149). This approach also minimizes the risk of random viral integration associated with conventional lentiviral methods and allows for better control over CAR-T cell function *in vivo.*

Such engineering advances are redefining the boundaries of cellular immunotherapy by enabling precise genetic programming of CAR-T cells to resist immunosuppression, enhance persistence, and reduce exhaustion. These next-generation platforms pave the way for safer, more effective applications of CAR-T therapy, especially in the context of solid tumors.

## 5 New technologies driving personalized CAR-T therapy

Traditional CAR-T cell development has relied heavily on trial-and-error approaches, which require manual analysis of thousands of tumor surface molecules. The structural complexity of CAR constructs including components such as single-chain variable fragments (scFv), linkers, transmembrane domains, and intracellular signaling modules further complicates the prediction of optimal therapeutic designs (150). Additionally, tumor heterogeneity poses a substantial challenge, particularly in solid tumors, where consistent and specific antigen targeting is difficult to achieve.

With the advent of artificial intelligence (AI), many of these limitations are being addressed (151). AI-driven platforms have increasingly been used in drug design, accelerating development timelines, and enhancing precision (152).

### 5.1 Novel target identification and validation

AI-based tumor-specific antigen discovery is central to the personalized CAR-T paradigm. AI models integrate large-scale datasets from cancer genomics, transcriptomics, and proteomics to identify ideal targets that are highly expressed in tumors but minimally expressed in normal tissues (152). Machine learning algorithms particularly XGBoost and Random Forest—have become standard tools in predictive modeling for antigen selection (153, 154).

In parallel, emerging studies have revealed the regulatory roles of non-coding Ribonucleic Acids (RNAs) in CAR-T functionality. Notably, circular RNAs (circRNAs) have been reported to influence CAR-T cell persistence, exhaustion, and efficacy in solid tumors, suggesting their potential as targets or biomarkers in RNA-based CAR-T optimization strategies (155).

In a recent study, a technique known as Time-lapse Imaging Microscopy in Nanowell Grids (TIMING) was developed to track CAR-T cell interactions with tumor cells at the single-cell level. By integrating single-cell transcriptomic data, researchers identified a CD8^+^ T cell subset termed CD8-fit cells with high migratory capacity and sustained cytotoxic activity. Notably, this subset was enriched specifically in patients who responded to CAR-T therapy, indicating its potential as a predictive biomarker (156).

The combination of single-cell RNA sequencing and AI-powered analytics allows for the precise identification of highly active T cell subpopulations, enhancing CAR-T efficacy while minimizing relapse risk.

### 5.2 AI-accelerated optimization of CAR-T manufacturing

Traditional CAR-T manufacturing processes rely on viral vectors, which are associated with low efficiency and potential genotoxicity risks (157). A team led by Zhang improved CAR-T production efficiency by 20-fold by modulating electroporation buffer osmolarity to inhibit activation of the cGAS–STING pathway, a key sensor of cytosolic DNA (158). This finding suggested that AI could be employed to optimize buffer parameters and predict their effects on intracellular signaling, thereby reducing experimental cycles, and improving manufacturing consistency.

## 6 Discussion

Tumor cells escape immune detection by downregulating MHC-I expression or disrupting interferon signaling, while metabolic factors such as lactic acidosis and hypoxia—along with suppressive immune cells like Tregs and MDSCs, further impair T cell function. To address this “dual barrier,” novel technologies have demonstrated strong synergistic potential (159, 160). For example, personalized messenger RNA (mRNA) vaccines guided by single-cell sequencing can elicit robust T cell responses against tumor-specific neoantigens, thereby reversing immune evasion (161). Meanwhile, nanoparticles loaded with lactate dehydrogenase inhibitors can locally neutralize the acidic TME and restore T cell metabolic activity. Particularly noteworthy are bispecific antibodies, such as PD-L1/CD3 (Bispecific T-cell Engagers) BiTEs, which simultaneously relieve immune suppression and redirect T cells toward tumor killing, achieving dual functional enhancement in solid tumors (162). These multi-target, multi-mechanism approaches may become a core strategy for overcoming resistance.

This review systematically outlined sophisticated CAR-T technologies to redefine the therapeutic boundaries of immunotherapies. The integration of cell therapies such as CAR-T cells with checkpoint blockade has yielded promising clinical outcomes. For example, “armored” CAR-T cells engineered to co-express PD-1 antibodies not only prevent T cell exhaustion but also remodel the tumor microenvironment through targeted cytotoxicity (163). Advances in gene editing have enabled the design of externally controllable or environment-responsive CAR-T systems, significantly reducing off-target toxicity (164). Furthermore, the development of universal CAR-T platforms and automated manufacturing technologies is improving scalability and accessibility while reducing production costs (165).

Despite these advances, several translational challenges remain. First, off-target effects from gene editing and risks associated with CAR-T clonal expansion require refined control strategies, such as base editing or epigenetic silencing (166, 167). Second, the high cost of personalized therapies highlights the urgent need for efficient biomarker screening systems and standardized manufacturing pipelines. For example, dynamic monitoring using circulating tumor DNA (ctDNA) could provide real-time evaluation of therapeutic efficacy and guide treatment adjustments. Lastly, successful clinical translation demands deep collaboration between academia and industry to establish an end-to-end ecosystem spanning from basic research to clinical application (168, 169).

Future efforts should focus on optimizing multi-level combination strategies. Integrating ICB with metabolic modulation and cell therapy may enable a transition from local tumor eradication to systemic immune reconstitution. Additionally, the deep integration of AI and multi-omics technologies will accelerate personalized treatment design for instance, using transfer learning algorithms to predict patient-specific resistance mechanisms and construct tailored therapeutic combinations. It is also essential to address ethical and equity concerns. Risk assessment in gene therapy, data privacy protection, and equitable resource allocation must advance in parallel with technological innovation.

In addition to CAR-T therapies, alternative engineered immune cells such as CAR-NK and CAR-M are emerging as promising modalities. CAR-NK cells offer innate cytotoxicity, reduced risk of graft-versus-host disease, and potential for allogeneic “off-the-shelf” production, addressing several limitations of autologous CAR-T therapies (170). Meanwhile, CAR-M cells macrophages modified to express chimeric antigen receptors can directly phagocytose tumor cells and modulate the tumor microenvironment through antigen presentation and pro-inflammatory cytokine release (78). These non–T cell-based strategies broaden the landscape of adoptive cell therapy and may complement or even synergize with CAR-T approaches in future combinatorial designs.

**Figure 1:**
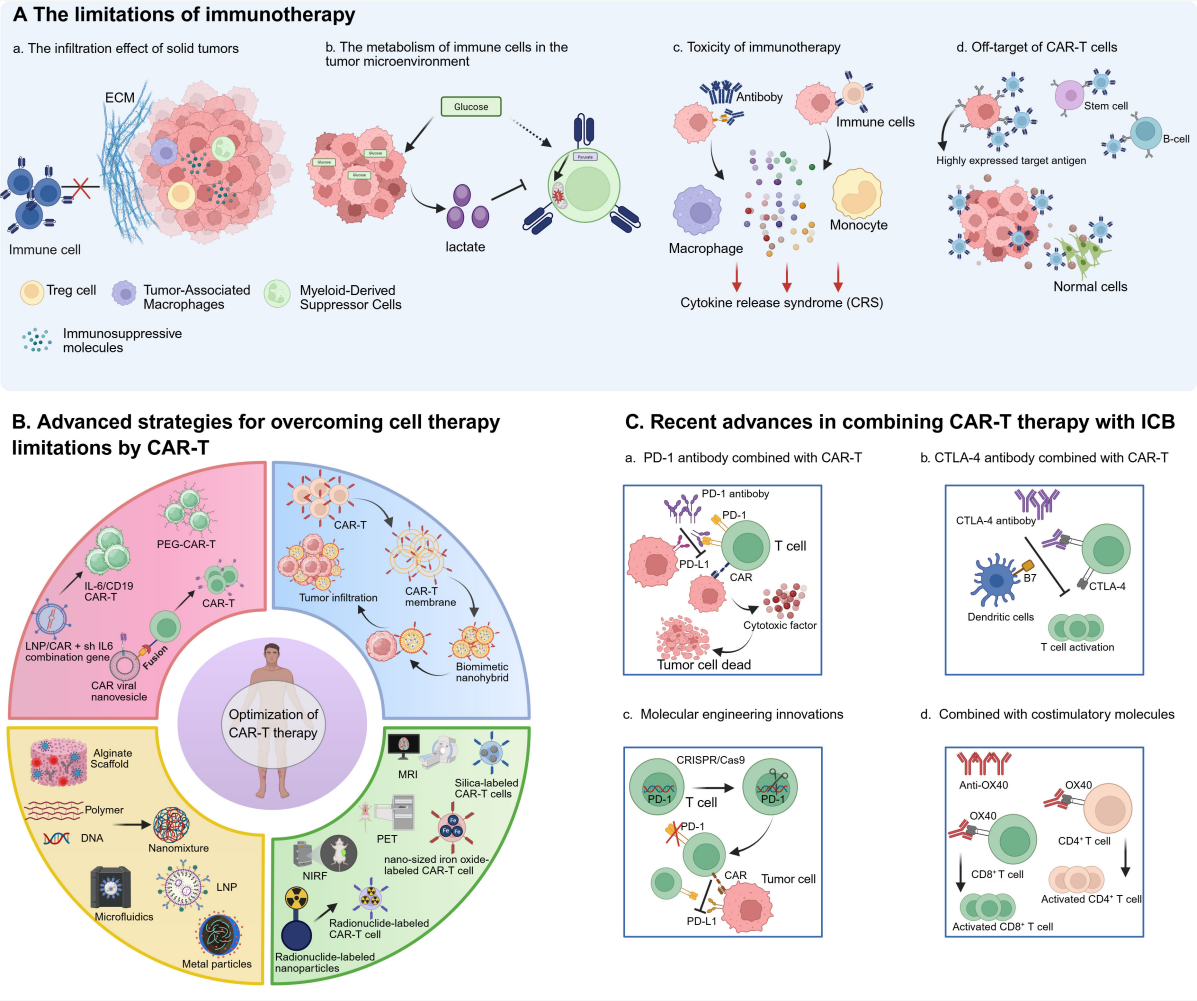
Schematic drawing of challenges and Improvements of immunotherapy. **(A)** The limitations of immunotherapy. **(a)** The infiltration effect of immune cells on solid tumors is limited due to the extracellular matrix and immunosuppression tumor microenvironment. **(b)** In the tumor microenvironment, tumor cells consume excessive glucose and produce more lactic acid, which affects the activity of immune cells. **(c)** After immune cells and antiboby bind to tumor cells, they produce cytokines and feed back to macrophages and monocytes for excessive production of cytokines, resulting in CRS. **(d)** Immune cells can recognize normal cells with low expression of target antigens, resulting in off-target side effects. **(B)** Advanced strategies for overcoming cell therapy limitations by CAR-T: bioengineered CAR-T cells have been designed with biomimetic enhancements to promote infiltration into solid tumors; nanocarrier-based platforms have been leveraged to streamline CAR-T cell manufacturing, reducing production costs and improving scalability; imaging-integrated nanoparticles have been combined with CAR-T cells to enable real-time monitoring of their activity, trafficking, and biodistribution; and nanodelivery systems have been engineered to precisely modulate cytokine release syndrome (CRS) and neurotoxicity by controlling inflammatory mediator levels. **(C)** Recent advances in combining CAR-T therapy with ICB. **(a)** The combination of PD-1 antiboby and CAR-T cells can reduce tumor immunosuppression and enhance the activity of CAR-T cells. **(b)** The combination of CTLA-4 antibody and CAR-T cells can enhance the persistence of CAR-T cells and promote their proliferation. **(c)** The combination of CAR-T and OX40 stimulating molecules increased the activation and proliferation of T cells. **(d)** The combination of CAR-T therapy with the costimulatory molecule OX40 enhances T cell activation and proliferation.

Only through multidimensional collaboration and innovation can immunotherapy truly overcome resistance and benefit a broader patient population (**Figure 1**).
